# Comparing the performance of a deep learning-based lung gross tumour volume segmentation algorithm before and after transfer learning in a new hospital

**DOI:** 10.1093/bjro/tzad008

**Published:** 2023-12-12

**Authors:** Chaitanya Kulkarni, Umesh Sherkhane, Vinay Jaiswar, Sneha Mithun, Dinesh Mysore Siddu, Venkatesh Rangarajan, Andre Dekker, Alberto Traverso, Ashish Jha, Leonard Wee

**Affiliations:** Philips Research, Philips Innovation Campus, Bengaluru, Karnataka 560045, India; Department of Radiation Oncology (Maastro), GROW School for Oncology and Reproduction, Maastricht University Medical Centre+, Maastricht 6229 ET, The Netherlands; Department of Radiation Oncology (Maastro), GROW School for Oncology and Reproduction, Maastricht University Medical Centre+, Maastricht 6229 ET, The Netherlands; Department of Nuclear Medicine and Radiology, Tata Memorial Hospital Mumbai, Mumbai, Maharashtra 400012, India; Department of Nuclear Medicine and Radiology, Tata Memorial Hospital Mumbai, Mumbai, Maharashtra 400012, India; Department of Radiation Oncology (Maastro), GROW School for Oncology and Reproduction, Maastricht University Medical Centre+, Maastricht 6229 ET, The Netherlands; Department of Nuclear Medicine and Radiology, Tata Memorial Hospital Mumbai, Mumbai, Maharashtra 400012, India; Philips Research, Philips Innovation Campus, Bengaluru, Karnataka 560045, India; Department of Nuclear Medicine and Radiology, Tata Memorial Hospital Mumbai, Mumbai, Maharashtra 400012, India; Department of Radiation Oncology (Maastro), GROW School for Oncology and Reproduction, Maastricht University Medical Centre+, Maastricht 6229 ET, The Netherlands; Department of Radiation Oncology (Maastro), GROW School for Oncology and Reproduction, Maastricht University Medical Centre+, Maastricht 6229 ET, The Netherlands; Faculty of Medicine, University Vita Salute, San Raffaele Hospital, 20132 Milan, Italy; Department of Nuclear Medicine and Radiology, Tata Memorial Hospital Mumbai, Mumbai, Maharashtra 400012, India; Department of Radiation Oncology (Maastro), GROW School for Oncology and Reproduction, Maastricht University Medical Centre+, Maastricht 6229 ET, The Netherlands

**Keywords:** deep learning, automatic segmentation, lung cancer, gross tumour volume, transfer learning, computed tomography, radiotherapy

## Abstract

**Objectives:**

Radiation therapy for lung cancer requires a gross tumour volume (GTV) to be carefully outlined by a skilled radiation oncologist (RO) to accurately pinpoint high radiation dose to a malignant mass while simultaneously minimizing radiation damage to adjacent normal tissues. This is manually intensive and tedious however, it is feasible to train a deep learning (DL) neural network that could assist ROs to delineate the GTV. However, DL trained on large openly accessible data sets might not perform well when applied to a superficially similar task but in a different clinical setting. In this work, we tested the performance of DL automatic lung GTV segmentation model trained on open-access Dutch data when used on Indian patients from a large public tertiary hospital, and hypothesized that *generic* DL performance could be improved for a specific *local* clinical context, by means of modest transfer-learning on a small representative local subset.

**Methods:**

X-ray computed tomography (CT) series in a public data set called “NSCLC-Radiomics” from The Cancer Imaging Archive was first used to train a DL-based lung GTV segmentation model (Model 1). Its performance was assessed using a different open access data set (Interobserver1) of Dutch subjects plus a private Indian data set from a local tertiary hospital (Test Set 2). Another Indian data set (Retrain Set 1) was used to fine-tune the former DL model using a transfer learning method. The Indian data sets were taken from CT of a hybrid scanner based in nuclear medicine, but the GTV was drawn by skilled Indian ROs. The final (after fine-tuning) model (Model 2) was then re-evaluated in “Interobserver1” and “Test Set 2.” Dice similarity coefficient (DSC), precision, and recall were used as geometric segmentation performance metrics.

**Results:**

Model 1 trained exclusively on Dutch scans showed a significant fall in performance when tested on “Test Set 2.” However, the DSC of Model 2 recovered by 14 percentage points when evaluated in the same test set. Precision and recall showed a similar rebound of performance after transfer learning, in spite of using a comparatively small sample size. The performance of both models, before and after the fine-tuning, did not significantly change the segmentation performance in “Interobserver1.”

**Conclusions:**

A large public open-access data set was used to train a generic DL model for lung GTV segmentation, but this did not perform well initially in the Indian clinical context. Using transfer learning methods, it was feasible to efficiently and easily fine-tune the generic model using only a small number of local examples from the Indian hospital. This led to a recovery of some of the geometric segmentation performance, but the tuning did not appear to affect the performance of the model in another open-access data set.

**Advances in knowledge:**

Caution is needed when using models trained on large volumes of international data in a local clinical setting, even when that training data set is of good quality. Minor differences in scan acquisition and clinician delineation preferences may result in an apparent drop in performance. However, DL models have the advantage of being efficiently “adapted” from a generic to a locally specific context, with only a small amount of fine-tuning by means of transfer learning on a small local institutional data set.

## Introduction

Lung cancer (LC) is the leading cause of cancer-related deaths according to 2020 global cancer statistics, with an age-sex adjusted mortality of 18 out of every 100 000 persons.^1^ Non-small cell lung cancer (NSCLC) makes up 80% of all LC cases. Radiation therapy (RT), either alone or in combination with systemic therapies (chemotherapy, immunotherapy), plays a major role in reducing risk of mortality in advanced stage NSCLC. Accurate RT requires a GTV to be carefully outlined on x-ray computed tomography (CT) scans by a skilled radiation oncologist (RO), such that a high radiation dose can be directed to a malignant mass while simultaneously minimizing radiation damage to adjacent normal tissues. Manual outlining is not only time-consuming and tedious, but it tends to be highly dependent on each RO’s clinical experience and subjective style, thus leading to inter-observer differences in the delineated GTV.^2^^,^^3^

The application of artificial intelligence (AI) across a broad spectrum of radiology and RT workflows has the potential to dramatically save time and reduce unjustified deviation between skilled operators, however, this promise has not yet been widely used for delineating the GTV. Deep learning neural networks (in short, “deep learning”—DL) for GTV delineation denotes a specific form of AI algorithm that requires a vast number of “training examples” from expert ROs as input to iteratively adjust its internal state, then said algorithm is able to mimic (to a measurably extent of accuracy) the delineation performance of a trained RO. Furthermore, accurate localization of a GTV is a pre-requisite step in quantitative image-based biomarker analysis (radiomics) where the aim is to improve prognostic/diagnostic performance when making clinical decisions.

A number of GTV segmentation approaches have been tested, including atlas-based, active contour, and mixture-based models.^4–6^ Recently, the focus has switched to DL-based UNET^7^ and closely related variants, such as attention-UNET^8^ and UNET++.^9^ Recent DL-based results appear very promising, for instance, Primakov et al^10^ with a 2D-based method developed and validated in 1328 thoracic CT scans from 8 institutions.

While training of DL requires large data sets, data availability is gradually becoming less of a barrier, with several contributions of open access GTV-annotated data sets such as NSCLC-Radiomics^11–13^ for LC radiotherapy planning CTs and LIDC-IDRI^14^ for lung nodules in diagnostic CTs. Additionally, DL methods have steadily become more efficient regarding sample size, and data augmentation techniques have been used to achieve a wider degree of generalizability.

However, the major obstacle for clinical acceptability of automated GTV segmentation is that one source of truth does not exist; there is no fully unequivocal binary segregation of a GTV from surrounding lung tissue. Every RO-generated training example for DL is therefore (to some degree) subjective and operator-dependent. Anecdotally, we expect some convergence among ROs who work together, but studies show that the boundaries of a GTV delineation generally vary among observers,^15^^,^^16^ and there might be particular aspects of “style” (preference) unique to a given RO and/or institution. Differences among CT scanners and image acquisition protocols (eg, contrast/non-contrast) may further exacerbate clinical context sensitivity.

This raises an important question for LC GTV delineation because a DL model trained using data in one RT clinic by one or more of their ROs might not likely to be clinically acceptable to another RT clinic among a different group of ROs. The findings of Primakov et al suggests that statistical noise in clinical studies arises from unwanted divergence between observers (a precision question), but on the other hand each RO is also medically responsible for defining the *correct* extent of the tumour in front of them with each and every individual case (an accuracy question).

Our present hypothesis is that a pre-trained DL model for LC GTV delineation—developed using an open access Dutch radiation oncology data set—can be efficiently re-trained to make a better adaptation of LC GTV delineation for an Indian hospital, using only a relatively small sample size from the Indian clinical setting. We propose this as a specifically localized and limited application of “transfer learning,” where an algorithm trained on a specific data set is adapted to a similar task in a distinct clinical context with different image acquisition settings, by updating only a part of the aforementioned pre-trained model.

## Methods

### Data sets

The open access data set “NSCLC-Radiomics”^11–13^ hosted on The Cancer Imaging Archive (TCIA; www.cancerimagingarchive.net) was used to pre-train an LC GTV delineation DL model (hereafter Model 1). The training cohort consists of 422 NSCLC patients’ RT planning CT from a single Dutch institution, with or without intravenous contrast enhancement, plus an LC GTV outline provided by an expert RO. One subject was missing an LC GTV due to surgical resection of a lung, therefore, only 421 cases were usable for DL model development. The CT scans and LC GTV delineation were obtained by a single RT clinic for the purpose of routine RT treatment, and were used entirely “as is” for this work; no re-editing of delineations were performed.

Another TCIA collection “Interobserver1” was kept as a constant test set.^12^^,^^13^^,^^17^^,^^18^ We used 20 patients that had been diagnosed with surgically operable NSCLC (but were not intended for RT) and were scanned in a CT by a university hospital diagnostic radiology department. On each CT volume, an LC GTV delineation was independently and separately performed by 5 expert ROs from the formerly mentioned RT clinic. Thus, the “Interobserver1” set comprises CT scans from a wholly independent scanner and clinical workflow, but the delineation of LC GTV was nominally consistent with the above-mentioned training set.

**Figure 1.**
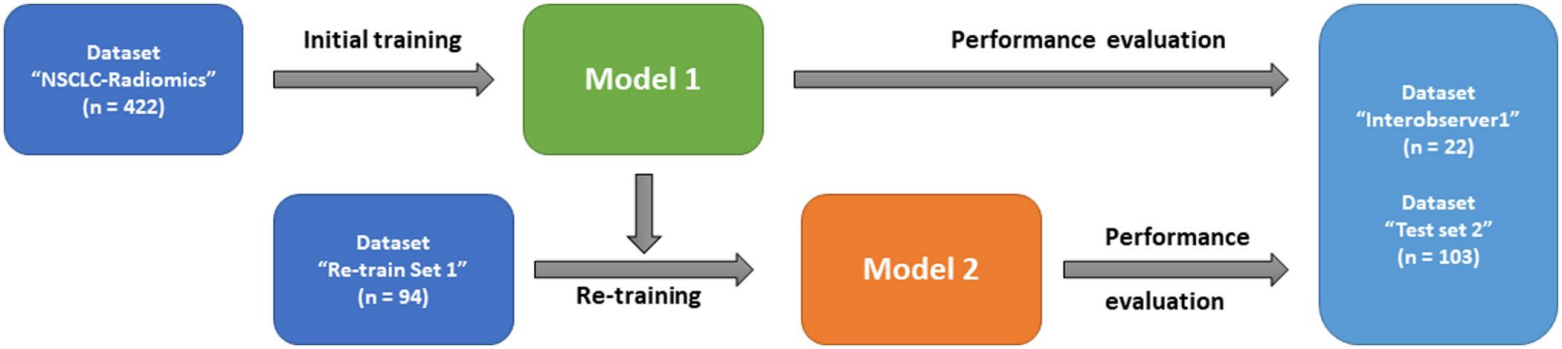
Schematic illustrating how 2 distinct data sets were used for training (NSCLC-Radiomics) of the initial generic model and then an Indian data set (Re-train Set 1) was used for re-training of a locally adapted model. Both models were then evaluated for performance in terms of Dice Similarity Coefficient, Recall, and Precision in 2 other independent data sets that were “not” used during the initial training and the re-training.

For the purpose of re-training the DL model, we obtained full-body CT scans from a hybrid PET-CT device housed in one of the largest public nuclear medicine departments in India. There were 94 subjects in this set known as “Re-train Set 1.” A distinct set of 103 hitherto unseen Indian subjects were held out for performance evaluation as “Test Set 2.” All Indian patients had been diagnosed with NSCLC and LC GTV delineations were provided by an expert RO from the adjoining radiotherapy department of the same hospital. The “Re-train Set 1” used to partially re-train the previously developed lung GTV segmentation. “Interobserver1” and “Test Set 2” were used to independently check the performance of GTV segmentation before as well as after the partial retraining. To help the reader understand this procedure flow, we summarize the aforementioned data sets in a schematic (see Figure 1).

### Pre-processing

Each CT volume was converted from DICOM to NIfTI (nii) format using the python library *dicom2nifti*.^19^ The corresponding LC GTV ground truth delineations as (DICOM) RTSTRUCT objects were also converted to nii format using the package *dicomrtstruct2nii*.^20^ A window of [−150, +250] HU was applied to set the brightness and contrast of the tissue of interest. Intensity values in each image was scaled as min-max normalization was applied. There was no voxel resampling/interpolation within the axial plane or between slices. Each pre-processed slice of a given CT study was exported as a single nii file together with its matching binary mask (1 inside the LC GTV and 0 elsewhere) nii file.

### Deep learning network architecture

The DL architecture we used was an implementation of an “Attention UNET”^8^ that has been successfully applied to other medical image segmentation tasks.^8^^,^^21^ The network has been modified by (1) adding a batch normalization layer at the start of every convolutional block to normalize the input, and (2) simplified to a 2D segmentation network (that is, we process CT series slice by slice instead of the full 3D volume at once).

A schematic diagram of the network is shown in Figure S1. The size of the input and output layers have been fixed to the standard dimensions of our axial CT images (512 pixels by 512 pixels). A basic “block” of the network comprised 1 batch normalization layer, 2 convolutional layers, and 1 max-pooling layer. There were 9 blocks with 4 blocks each along the downsizing and upsizing sides of the UNET, respectively, and one block comprising the bottleneck. The convolution filter was 3x3 pixels in size, with stride of 1 and padding of 1. We used a leaky rectified linear unit (ReLU) with alpha of 0.1 for activation, and an ADAM optimizer with learning rate 10^−6^, and Dice Loss (ie, complement of the Dice similarity index) as the cost function. The output layer comprised a single convolution layer, with a single filter of size 1x1 pixels and a sigmoid activation function to produce a segmentation mask.

Additional details of the attention-merge gates are given in Figure S2. The purpose of integrating attention gates into a UNET structure was to combine target object localization at each level of the UNET with subsequent segmentation in the next level down, so as to enhance sensitivity to target pixels while suppressing response to non-target pixels, at the same time also making more efficient use of the training samples.

### Model training and validation

We pre-trained an LC GTV model using 421 subjects in the NSCLC-Radiomics data set for a total of 300 epochs and a batch size of 6 (Model 1). During training, 10% of the subjects was kept apart for internal validation and model tuning. This initial model was then transfer-trained, keeping the pre-trained weights as the initial starting condition, however using only the “Re-train Set 1” from the Indian hospital for a maximum of 150 epochs also with a batch size of 6 (thus resulting in Model 2). This was done to guard against “catastrophic forgetting” where the pre-trained model suddenly over-fits to the new data set thereby losing all of its former training. All models were trained on a single NVIDIA GPU device using standard libraries (TensorFlow 2.0, CUDA 10.0, and CUDNN 7.4). Computations were performed locally inside the computing environment of the participating Indian hospital using anonymized patient data.

### Model evaluation

Before (Model 1) and after (Model 2) the re-training, both GTV segmentation models were independently tested using the public open access “Interobserver1” data set from The Netherlands and “Test Set 2” from the Indian hospital. The geometric accuracy of the model was evaluated using the Dice similarity coefficient (DSC), Recall, and Precision. A schematic diagram illustrating how the performance metrics of DSC, Recall, and Precision are computed is shown in Figure S3.

### Statistical analyses

In the “Interobserver1” evaluation data set, we obtained a scatter plot of DSC for each subject by comparing ROs pair-wise against each other (10 possible combinations). We compared Model 1 and Model 2, respectively, against each RO (5 possible combinations). One-way analysis of variance (ANOVA) test for DSC against 3 LC GTV segmentation methods (first—manually by ROs, next—by Model 1, and finally—by Model 2) was performed, followed by *post hoc* Tukey’s Honestly Significant Differences (HSD) statistical test for pair-wise methods. The same analysis was applied to Precision and Recall. The local Indian data set “Test Set 2” only had a single RO delineation of each LC GTV, however, the same analysis for DSC, Precision, and Recall was performed.

Additionally, in the “Interobserver1” data set, we can obtain 5 masks of the same LC GTV from 5 different ROs. Overlapping these 5 LC GTV outlines will provide us 5 different regions; the first region being the area where *all 5* ROs marked as LC GTV, second region being where *4 or more* RO marked as LC GTV and so on, and the final regions where *at least* one RO marked as LC GTV. To understand the percentage of the mask the algorithm was able to segment, we used Recall as the performance metric for statistical analysis.

## Results

Table 1 shows the clinical characteristics of the subjects in each of the 4 data sets. The subjects in “NSCLC-Radiomics” and Indian “Re-train Set 1” have a median age greater than 55, and both data sets contain significantly more males than females. In “NSCLC-Radiomics,” the majority of cases were Stage III but no stage IV; in the data as-retrieved from TCIA, there appears to be a typographical error since at least 1 of the subjects is M1. We kept these, and any other unknown/missing stages, in the study, since the subject’s CT was available and this was not expected to adversely affect the segmentation performance of the model. This is quite different from the data collected in the Indian hospital, with the majority of cases being stages III and IV. Histology appears quite diverse in the public data sets; however, Indian subjects primarily were adenocarcinoma and squamous-cell carcinoma.

**Table 1.** Patient characteristics in the data used for the pre-trained and transfer learning models.

	NSCLC-Radiomics (initial training)	Indian hospital (retrain set 1)	Indian hospital (test set 2)	Interobserver1 (open access test set)
**Sample size**	**422**	**94**	**103**	**22**
**Median age (range)**	69 (34-92)	58 (22-77)	56 (30-83)	Not recorded
**Biological sex**				
Female	132	32	32	9
Male	290	62	71	13
**T stage**				
T1	93	6	13	3
T2	156	48	48	17
T3	53	25	23	0
T4	117	15	19	2
Missing	3	0	0	0
**N stage**				
N0	170	29	47	3
N1	23	12	14	1
N2	141	46	37	17
N3	85	7	5	1
Missing	3	0	0	0
**M stage**				
M0	417	68	80	22
M1	1	26	23	0
Missing	4	0	0	0
**Overall stage**				
I	93	2	6	3
II	40	33	37	1
III	288	21	22	18
IV	0	38	38	0
Missing	1	0	0	0
**Histology**				
Adeno	51	75	83	10
Large cell	114	0	0	3
Squamous	152	17	15	6
Other	63	2	5	3
Unknown	42	0	0	0

Table 2 indicates the CT image acquisition settings as given by the DICOM header attributes. The scans for “NSCLC-Radiomics” were acquired from 2 Siemens CT scanners, whereas the Indian data sets were acquired from a Philips Gemini hybrid PET-CT scanner. The tube voltages and pixel resolution for the DICOM images were similar across all data sets. The reconstructed slice thickness for “NSCLC-radiomics” and “Interobserver1” were 3 and 5 mm, respectively, whereas the slice thickness varied patient-to-patient in the Indian data sets. The median tube current used in the Indian hospital scans was about factor three higher than open access data sets. A head-first-supine orientation was uniformly used in “NSCLC-Radiomics” and “Interobserver1” data set but the patient orientation could be either head-first or feet-first supine in the Indian data sets.

**Table 2.** DICOM header attributes of the CT used, with median and range of each property.

	NSCLC-Radiomics (train only)	Re-Train Set 1 (re-train only)	Test Set 2 (Indian test set)	Interobserver 1 (public test set)
**Sample size**	**422**	**94**	**103**	**22**
**Vendor(s)**	Siemens	Philips	Philips	Siemens
**Model(s)**	Biograph & Sensation	Gemini TF/TOF	Gemini TF/TOF	Not recorded
**Tube voltage**				
Median	120	120	120	Not recorded
Range	(120-140)	(110-140)	(110-140)	
**Tube current**				
Median	80	268	287	Not recorded
Range	(40-552)	(80-463)	(40-463)	
**Pixel spacing**				
Median	0.98	0.98	0.98	0.98
Range	(0.72-0.98)	(0.53-1.17)	(0.52-1.37)	(0.98-0.98)
**Slice thickness**				
Median	3	3.75	2	5
Range	(3-3)	(2-5)	(2-5)	(5-5)
**Pat. orientation**				
Head-first supine	422	64	65	22
Feet-first supine	0	30	38	0

**Figure 2.**
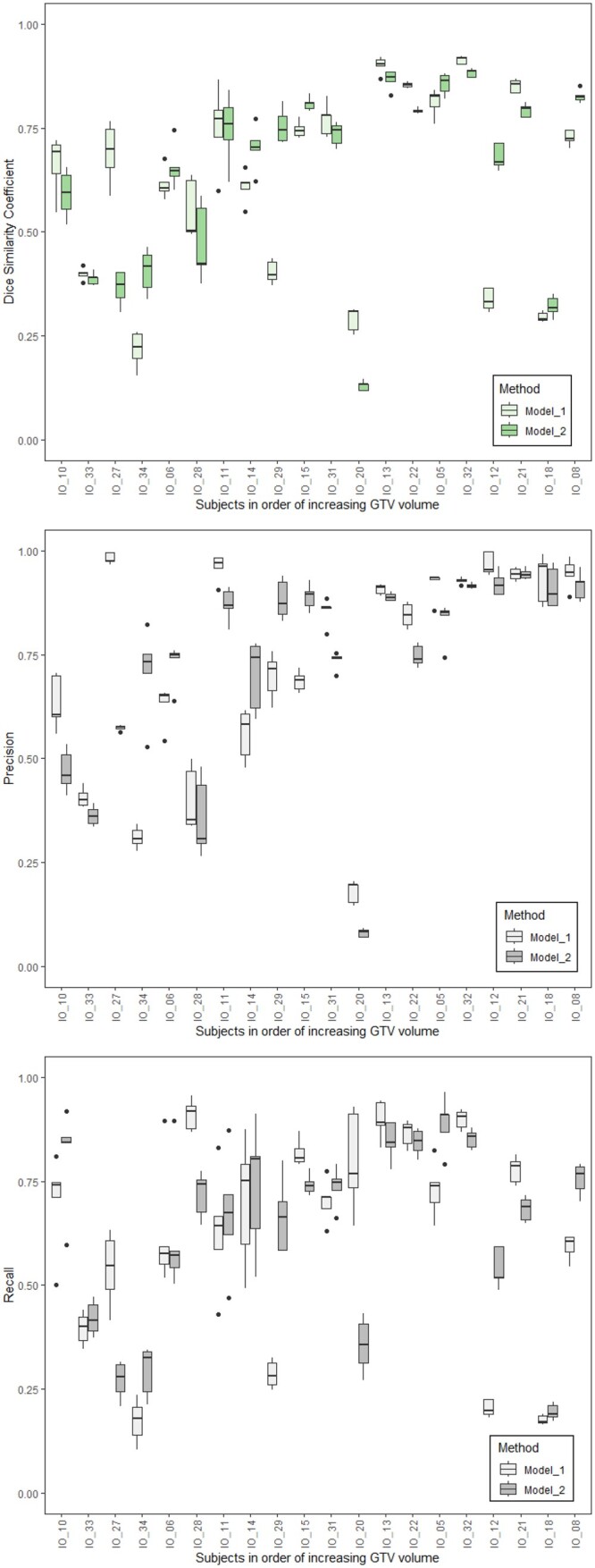
Subfigure (A) (at top) shows the distribution of the Dice score for Model 1 and 2. Subfigures (B) (middle) and (C) (bottom) show the distribution of precision and recall, respectively, for Model 1 and Model 2. Each metric has been computed with respect to the 5 RO manual LC GTV drawings per patient. The subjects have been ordered along the horizontal axis according to increasing LC GTV absolute volume. Abbreviation: GTV = gross tumour volume.

In the “Interobserver1” set, we observed the dispersion of DSC between ROs tended to be larger for the smaller tumours. In Figure 2A, the geometric agreement of LC GTV segmentation by Model 1 and by Model 2, compared relative to each RO, was visibly inferior to the ROs between themselves. The subject-by-subject DSC of Model 2 relative to Model 1 was mixed; in some subjects better results were observed, but worse in others. The ANOVA analysis indicated that inter-method differences were statistically significant (*P* < .001), and Tukey’s *post hoc* HSD showed that significant differences in DSC were between the human expert methods and the DL-based methods. The differences between Model 1 and Model 2 were not statistically significant (corrected *P*-value .79). The median (range) DSC according to method were: manual—0.84 (0.48-0.94), Model 1—0.68 (0.15-0.92), and Model 2—0.71 (0.12-0.89).

Results for Precision and Recall of Model 1 compared to Model 2 are shown in Figure 2B and C, respectively. The median (range) for Precision were: Model 1—0.86 (0.14-1.00) versus Model 2—0.79 (0.07-0.97). Likewise, median (range) for Recall were: Model 1—0.69 (0.10-0.96) versus Model 2—0.70 (0.17-0.96). Neither of these methods were statistically significantly different according to 1-way ANOVA.

Corresponding results for “Test Set 2” are given below in Table 3 indicating that the median and interquartile ranges appear to have changed favourably due to Model 2, but the absolute range (min-max) suggests that individual results remain highly heterogeneous. One-way ANOVA tests for DSC, Recall, and Precision suggests the differences between Model 1 and Model 2 for the former two are statistically significant, but not so for the latter.

**Table 3.** Dice, recall, and precision computed on Indian hospital test data for Model 1 and Model 2.

	DSC	Precision	Recall
**Model 1**			
Median (range)	0.63 (0.0-0.93)	0.83 (0.0-1.00)	0.58 (0.0-0.92)
Interquartile range	0.37-0.81	0.59-0.91	0.27-0.72
**Model 2**			
Median (range)	0.77 (0.0-0.93)	0.85 (0.0-0.97)	0.71 (0.0-0.94)
Interquartile range	0.56-0.85	0.73-0.91	0.46-0.84
**ANOVA test (Model 1 vs 2)**			
*P*-value	.01*	.15	.008**

ANOVA analysis indicates the difference in scores distribution for Model 1 and Model 2.

Abbreviation: DSC = dice similarity coefficient. *p < 0.05, **p < 0.01.

Table 4 shows the performance of the algorithm based on the number of ROs agreeing on the area of the LC GTV. The analysis was only done in “Interobserver1” for both Model 1 and Model 2. The Recall score for both models where all of the ROs marked an area as being a GTV was greater than 0.8. The Recall dropped to 0.65 and 0.50, if only one or two ROs drew an area as GTV, respectively. ANOVA analysis results shows a high degree of correlation between Recall scores of both models.

**Table 4.** Recall score computed based number of RO marking a certain area as a lesion.

	Recall				
	Area marked by all RO	Area marked by any 4 RO	Area marked by any 3 RO	Area marked by any 2 RO	Area marked by any 1 RO
**Model 1**					
Median (range)	0.85 (0.2-0.97)	0.77 (0.19-0.96)	0.73 (0.17-0.93)	0.65 (0.13-0.90)	0.50 (0.10-0.84)
Interquartile range	0.63-0.92	0.60-0.88	0.51-0.83	0.44-0.75	0.35-0.70
**Model 2**					
Median (range)	0.83 (0.2-0.99)	0.75 (0.21-0.95)	0.72 (0.20-0.92)	0.66 (0.18-0.86)	0.56 (0.16-0.77)
Interquartile range	0.59-0.91	0.57-0.86	0.52-0.81	0.46-0.77	0.42-0.66
**ANOVA test (Model 1 vs 2)**					
*P*-value	.96	.91	.87	.84	.84

Abbreviation: RO = radiation oncologist.

**Figure 3.**
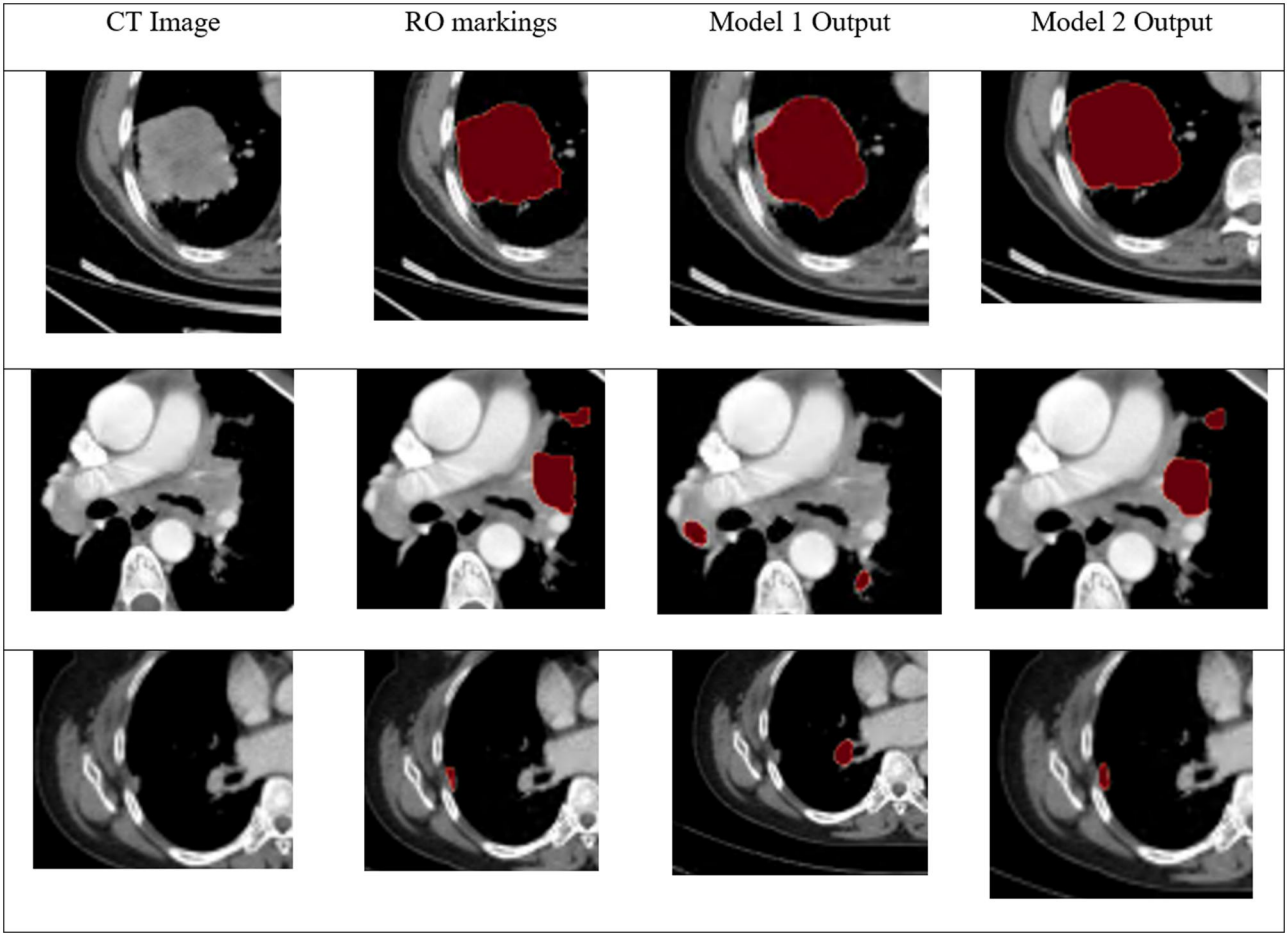
Three example subjects on Indian test set, along with markings from RO and the output from Model 1 and Model 2. Abbreviation: RO = radiation oncologist.

Figure 3 illustrates representative CT slices from 3 representative patients in the “Test Set 2” data set. In the first subject (top row), both Model 1 and Model 2 performed equally well and agreed reasonably with the RO drawing. In the second (middle row) and third (bottom row) subjects, Model 1 had clearly no overlap with the human expert-segmented LC GTV, however, Model 2 was in much better agreement with the RO’s drawing.

## Discussion

We confirmed the hypothesis that a generic GTV segmentation model that had been trained on a large amount of open access CT scans, using a transfer-learning method, can be efficiently fine-tuned on a relatively small local data set from the intended clinical context. This adaptation does not need a large amount of local data, and does not need massive computational resources (ie, this work was performed with a single GPU chip of modest specification). Our work supports the hypothesis that generic segmentation models that are pre-trained on large international data sets can be feasibly adapted into a local context, with their specific clinical requirements and own imaging equipment, thus lowering the barrier for data collection and model re-development within a local clinical setting.

We initially trained an Attention UNET DL (Model 1) for lung GTV segmentation, but observed that it did not perform as well in the Indian clinical context as in the Dutch one. There could be many possible reasons for this, however, we can speculate as regards the most influential sources of the divergence. First, the influence of scanner equipment, reconstruction parameters, and image acquisition settings. The metadata associated with “NSCLC-Radiomics” indicates axial CTs from vendor Siemens were obtained in a Dutch radiotherapy department for the specific purpose of RT dose planning, and has been re-used here as is, ie, the same as approved for routine clinical treatment and without any editing. The metadata associated with “Interobserver1” informs that these were obtained on a diagnostic radiology Siemens CT scanner. The Indian local axial CTs were obtained from full-body scans in a nuclear medicine department equipped with a hybrid Philips PET-CT scanner. While there are broadly overlapping parameters between all 4 scan sets, we pointed out in the results pertaining to Table 2 that there are likely to be divergences due to vendor, image quality, and reconstruction methods.

Secondly, the GTV in “NSCLC-Radiomics” were all prepared by one senior Dutch RO with 20 years of clinical specialization in LC RT. The lung GTV in “Interobserver1” was independently delineated by 5 ROs with diverse levels of clinical experience ranging from a few years of specialization in RT up to as much as 2 decades. The Indian cohorts—“Re-train Set 1” and “Test Set 2”—had lung GTVs delineated by a single highly experienced Indian RO. Obviously, we must not rule out inter-observer differences arising from personal clinician preferences and history of clinical training. Studies show that interobserver GTV disagreements persist, even when drawn by qualified ROs that work in the same RT clinic.^22^ Tumour segmentation is in a class of clinical problems that defy having a “single source of truth”—unlike pathological examination of a biopsy sample under a microscope. Additionally, patient demographics differ in the sense that Indian subjects are more likely to be admitted with already advanced or metastatic cancer, as seen in M1 versus M0 status in aforementioned Table 1. These results suggest that, while the learning and target domains seem superficially similar, there are still significant differences where a transfer-learning approach could be beneficial in regard to adapting a generic DL model to meet a specific local clinical context.

In Table 3, we observed that Model 1 (before fine-tuning) in the Indian “Test Set 2” data set had a median recall of 0.58, which indicated that in a majority of patients a portion of the lung GTV would not be correctly segmented by the model. After re-training (Model 2), the same recall index had improved to 0.71, a significant improvement according to analysis of variance (ANOVA). We focus here on the recall performance (see Figure S3 as reminder of definitions). Healthy tissue mis-segmented as part of a GTV would be easily corrected during routine radiotherapy plan review. However, if a portion of real tumour has been mis-segmented as being normal tissue and if this has also been overlooked by an RO, then tumoricidal radiotherapy dose would not be targeted to that location—the tumour will likely continue to grow and eventually metastasize. Thus, from a clinical perspective, we assumed that mis-segmentation of a portion of lung GTV would be more detrimental to the patient than a mis-segmentation of healthy lung tissue.

The implication of Table 4 is the automated DL-based segmentation generally segments a region in the “Interobserver1” data set where all 5 independent ROs consistently agree forms part of the lung GTV, with a median recall of 0.85 for Model 1 (before fine-tuning) and 0.83 for Model 2 (after fine-tuning). As the inter-observer discrepancy between ROs increases, we see also that the models perform more poorly. However, from a clinical perspective, we can infer that the models can more readily focus on the core of the GTV where all the ROs seemed quite certain, but then the model errors increase for the contentious/debatable edges of the GTV where the RO themselves tend to disagree among each other.

From an inter-observer variability study,^22^ we expect that independent manual delineation among expert clinicians has a Dice score between 0.7 and 0.8. In our work, a median Dice score around 0.7 suggests that our automated segmentation method still requires review and (if needed) manual adjustment by a skilled RO; we do “not” recommend our tool to be used during routine clinical radiotherapy unless rigorous quality assurance is conducted beforehand. However, we note that the workflow is not at all different from a manual segmentation if performed by a junior RO specialist or a clinician-in-training; all such work by a less-experienced human has to be checked, corrected, and verified by a highly experienced RO before being used in treatment.

We noted that updating the model using “Re-train Set 1” comprising less than 25% of “NSCLC-Radiomics” sample size, was already able to improve the performance in the Indian “Test Set 2” by 14 percentage points in median DSC. This indicates that, for re-training of an existing model, introducing adequate variation (different patients, different CT settings) is potentially more important than purely sample size by itself. At the same time, Model 1 (before fine-tuning) and Model 2 (after fine-tuning) did not change on the unseen “Interobserver1” data set. There is no special rule for 25% for the re-training sample size, but we note that it is consistent with widely used best practices in DL development, where a holdout data set of between 20% and 30% of the main data set is quarantined for model performance validation.

We note that fine-tuning a model using transfer learning methods on different data set always exposes some risk of “catastrophic forgetting,” such that Model 2 might lose all the training from “NSCLC-Radiomics” data set, especially if the new model had been excessively trained with the Indian data. We reduced this risk by carefully monitoring the training and internal validation loss curves, and intentionally stopped the re-training epochs if we saw any sign of over-fitting to the newly introduced data set. We found no evidence of catastrophic forgetting up to 150 epochs for this present work.

In literature to date, a deep-learning network such as UNET and its related variations have demonstrated state-of-the-art performance when given segmentation tasks in different contexts. A canonical UNET and the Attention UNET which we used in this work share a very similar overall structure, but the latter merges information from the decoding/down-sampling part of the “U” to the encoding/up-sampling part using a logic construct known as “attention” (see Figure S2). Oktay et al^8^ showed in their work that the inclusion of attention helps a UNET to reduce the number of false positive pixels in biomedical segmentation problems that contain background clutter and dense information within the image. It is inferred that attention works by telling a segmentation network to preferentially add weight to image features that are relevant to the segmentation, rather than consider features from everywhere within an image. Additionally, as distinct from a canonical UNET, our models include an additional batch CT intensity normalization step in front of every convolutional block to assist with model convergence (see again Figure S2).^23^

We intended that our methods would be feasible for smaller clinics in low- and middle-income countries to efficiently adapt generic DL models trained elsewhere to fit into their specific clinical context. Therefore, we intentionally kept our software footprint relatively small; the Attention UNET in this work was implemented as a 2D model; that is, segmentation of the lung GTV was performed slice-wise, ie, treating each axial CT images as if they were independent inputs. We acknowledge this is not explicitly true, because we know that information on immediately adjacent CT slices must have some amount of common anatomical information. This may not be optimal for all possible tumour segmentation contexts, since the information about the tumour and the anatomy on adjacent slices for the same patient might be potentially useful for the overall segmentation task. Variations to our model which are being considered in a future publication include colour-image segmentation (using a CT axial slices and its 2 immediate adjacent slices in the red-green-blue channels) and fully-3D segmentation, but these alternatives led to a large increase in computing requirements and therefore we did not add these to the present study.

We did not apply spatial interpolation as pre-processing on the input images, so we speculate that the present work should not be sensitive to interpolation or resampling artefacts. Since we segmented slice-wise, we did not have to apply post-processing to smooth the GTV segmentation result back to the original CT inter-slice spacing, which could also have introduced some unintentional artefacts. This robustness could be a potential advantage when working with data sets containing variable CT-reconstructed slice thicknesses.

### Limitations and future directions

We acknowledge that geometric agreement (Dice, Precision, and Recall) is merely weak surrogate for actual clinical acceptability for use in RT dose planning. We recommend that automated segmentation models should ideally be evaluated in a prospective double-blinded manner, also with multiple independent ROs, within the intended clinical context of use. As shown by Lustberg et al,^24^ the clinical acceptability of automated segmentation might initially be low, but significant time savings can be demonstrated by measuring the time taken to adjust an automatically generated segmentation as opposed to drawing an entire mask from scratch.

We have not yet performed an exhaustive simulation of how much new imaging data are required from the local clinical context in order to re-train a segmentation model according to transfer-learning methods. We are unable to state *a priori* exactly many epochs of training would be optimal for the fine-tuning, since this might change depending on task and on clinical context. We expect that the same approach should also work at yet another hospital, but we do not yet have results for this.

One useful direction of future work could be an unbiased estimation of model performance and optimization of the transfer training procedure, as a function of re-training data set size and other modifiable network training hyperparameters. Future studies might include robustly resampling the CT of arbitrary slice thickness to a uniform isotropic grid, and examining the potential benefits versus computational costs of a computationally more resource-intensive model. As mentioned above, there are other conditioning and attention approaches such as quasi-3D^25^ and patch-based^26^ alternatives for this class of 3D GTV segmentation problem.

Taking a broader view, we might also be able to more concretely define what clinical concerns about automated GTV segmentation that needs to be appropriately addressed with regards to technical feasibility, patient benefit, and cost-effectiveness of adapting large generic models for use within the Indian radiation oncology clinical setting.

## Conclusion

We initially developed an Attention UNET DL for automated LC GTV segmentation, but observed that it did not perform as well in the Indian clinical context as it did in The Netherlands. The initial model was fine-tuned using a small representative Indian data set using transfer-learning methods. This work showed that the segmentation performance significantly improved in hitherto unseen Indian test patients (Test Set 2) while at the same time the performance in a Dutch reference test data set (Interobserver1) did not change significantly. This fine-tuning was done with fewer than 100 Indian re-training cases and was intentionally stopped slightly early to reduce the risk of catastrophic forgetting. Thus, adaptation of large robust international GTV segmentation models to more closely match local clinical context is feasible with relatively small sample size and limited computing resources. This makes the use of advanced DL models readily attainable for smaller hospitals, even those in operating in low- or middle-income countries.

## Supplementary Material

tzad008_Supplementary_Data
