# Supplementary material for: Comparing the performance of a deep learning-based lung gross tumour volume segmentation algorithm before and after transfer learning in a new hospital
Source: BJR Open. 2023 Dec 12;6(1):tzad008. doi: 10.1093/bjro/tzad008 (PMC10860512; doi:10.1093/bjro/tzad008)
Supplement: tzad008_Supplementary_Data [file tzad008_supplementary_data.docx]

Supplementary Materials

A **deep learning neural network** is constructed from “blocks” of mathematical objects known as artificial neurons arranged in multiple “layers”. Inside each block, mathematical functions such as applying convolutional kernels, activation and down (or up) sampling is performed. A UNET is a specific architecture that comprises multiple layers in the encoding path matched with layers in the recoding path (see Figure S1 below) such as the input and output are the same dimensions in terms of number of pixels.


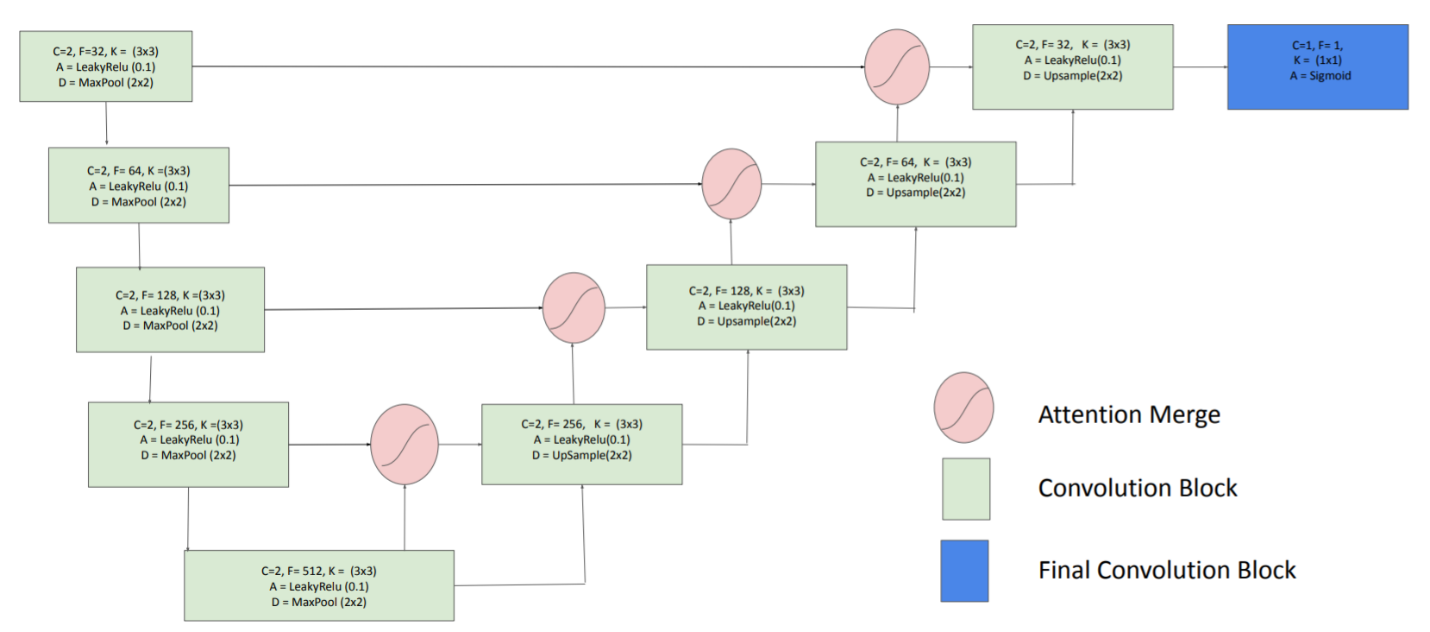


Figure S1: Lung cancer (LC) Gross tumor volume (GTV) deep learning neural network architecture illustrating the widely publicized UNET structure for image segmentation tasks. In the above figure C is the number of convolutional layers in each block, A denotes the activation function used, K is the convolution kernel size, F is the number of image filters in the convolutional layer and D is the dimensionality change function such as up-sampling to increase the size or max-pooling to decrease the size.

The **attention gates** are shown in Figure S2 receiving inputs from two convolutional blocks as shown as the red circles in Figure S1; one from the convolutional block in the encoding arm of the UNET (x) and the other from the matching convolutional block in the decoding arm of the UNET, with the same input dimensions (g). Each of these inputs is first passed through a convolutional layer, and the number of filters used varies from 256 to 32 for different attention blocks the number of filters for convolution is equal to half the number of filters used for the previous convolutional block (Fg) i.e. if the previous attention block uses 512 filters, the number of filters used in the attention block is 256. The kernel size for convolution used is 1x1 with valid padding and no activation. The output of the two convolutions is added and passed through a ReLU layer. The output of the ReLU layer is passed through another convolutional layer, this layer uses one filter and kernel size of 1x1, valid padding and sigmoid activation to generate an attention matrix. The attention matrix is multiplied with the previous layer output to provide attention merged output xl.


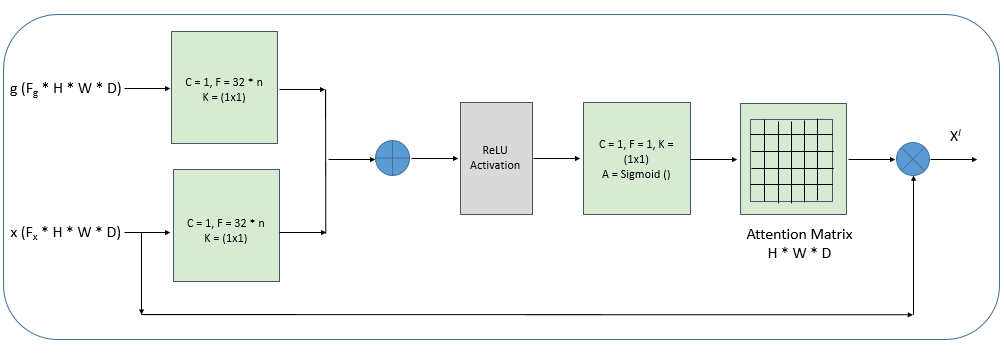


Figure S2: Illustration of additional details in the Attention Merge part in the previously mentioned UNET architecture. In the above figure, C indicates the number of convolutional layers, n is a number varying from 1 to 8 depending on where the attention layer is placed. x and g are the inputs and xl is the output of the attention merge. Fg and Fx are the number of filters from the previous layer and H, W and D (D=1 for our current problem) are the dimensions of the data at that layer.

**Geometric segmentation performance metrics** are based on a generalization of terminology such as true positive (TP), false positive (FP), true negative (TN) and false negative (FN) that are used for traditional diagnostic accuracy studies. For the task of geometric segmentation of objects in an image, the classifications above need to be applied pixel-wise across an entire image. Thus, the established nomenclature for segmentation performance in imaging is shown with the assistance of Figure S3.


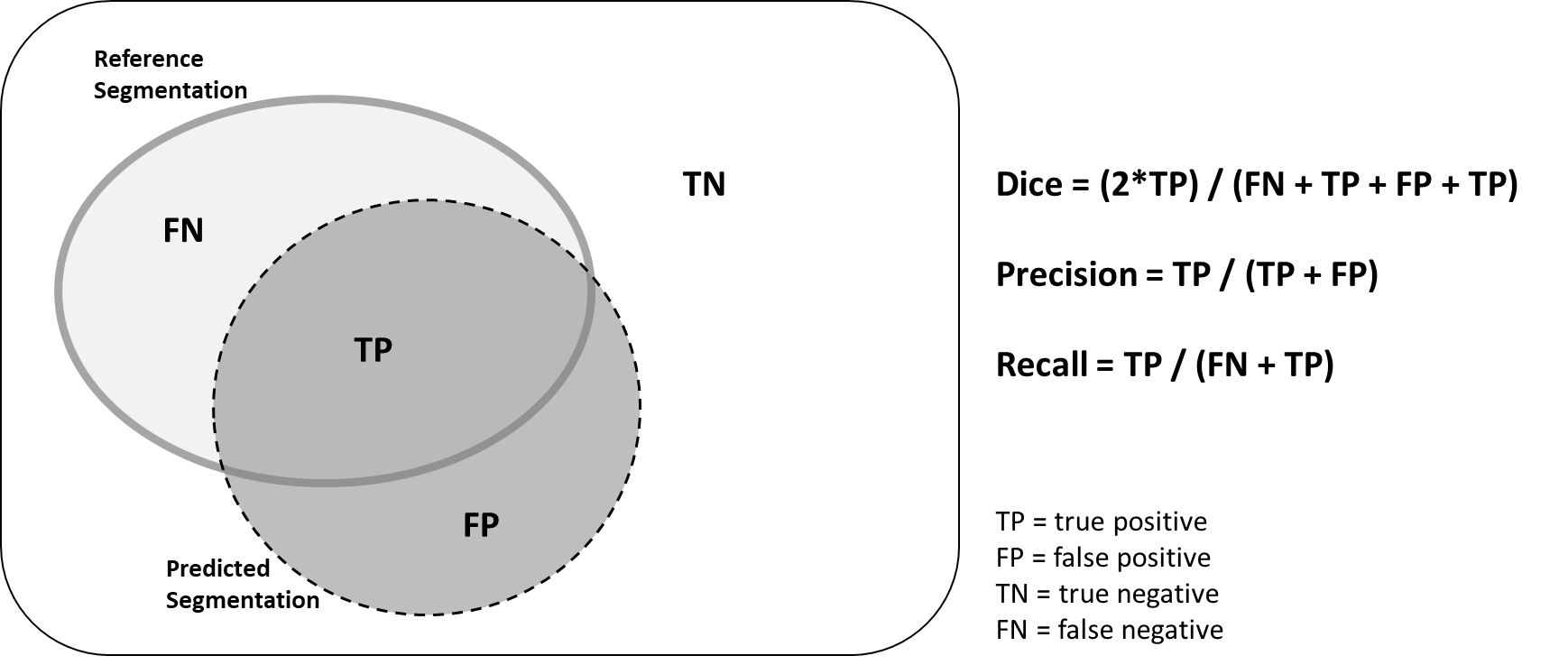


Figure S3: Definitions of Dice similarity (i.e. Dice for short), precision and recall, as applied to geometric segmentation of objects in images. The human expert reference segmentation of a fictional lung GTV is shown in the bold grey outline. The (predicted) segmentation generated by a deep learning model is shown in the thin dashed black outline. The intersection of human and model segmentation is known as TP. Non-tumor tissue included by the model is denoted FP. Tumor tissue missed by the model is known as FN. Regions where neither human expert nor model has included are defined as TN. The formulae for calculating Dice, Precision and Recall are indicated to the right hand side of the figure.
